# Osteomyelitis, Oxidative Stress and Related Biomarkers

**DOI:** 10.3390/antiox11061061

**Published:** 2022-05-27

**Authors:** Luca Massaccesi, Emanuela Galliera, Antonio Pellegrini, Giuseppe Banfi, Massimiliano Marco Corsi Romanelli

**Affiliations:** 1 Department of Biomedical Sciences for Health, Università degli Studi di Milano, 20133 Milan, Italy; emanuela.galliera@unimi.it (E.G.); mmcorsi@unimi.it (M.M.C.R.); 2 IRCCS Galeazzi Orthopaedic Institute, 20161 Milan, Italy; banfi.giuseppe@hsr.it; 3 Centre for Reconstructive Surgery and Osteoarticular Infections, IRCCS Galeazzi Orthopaedic Institute, 20161 Milan, Italy; antonio.pellegrini@grupposandonato.it; 4 Service of Laboratory Medicine1-Clinical Pathology, IRCCS Policlinico San Donato, San Donato Milanese, 20097 Milan, Italy

**Keywords:** osteomyelitis (OM), reactive oxygen species (ROS), oxidative stress (OS), oxidative stress biomarkers

## Abstract

Bone is a very dynamic tissue, subject to continuous renewal to maintain homeostasis through bone remodeling, a process promoted by two cell types: osteoblasts, of mesenchymal derivation, are responsible for the deposition of new material, and osteoclasts, which are hematopoietic cells, responsible for bone resorption. Osteomyelitis (OM) is an invasive infectious process, with several etiological agents, the most common being *Staphylococcus aureus*, affecting bone or bone marrow, and severely impairing bone homeostasis, resulting in osteolysis. One of the characteristic features of OM is a strong state of oxidative stress (OS) with severe consequences on the delicate balance between osteoblastogenesis and osteoclastogenesis. Here we describe this, analyzing the effects of OS in bone remodeling and discussing the need for new, easy-to-measure and widely available OS biomarkers that will provide valid support in the management of the disease.

## 1. Introduction

**Figure 1:**
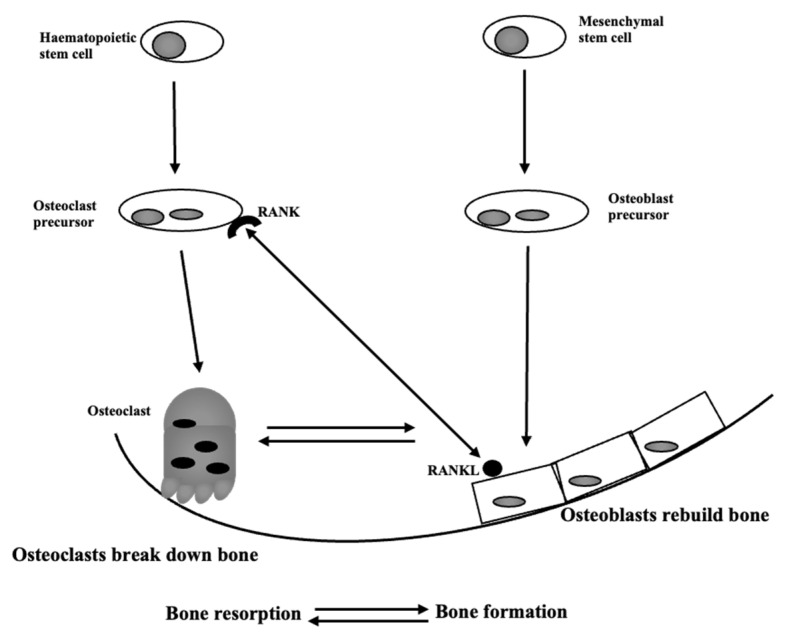
Maintenance of bone structure. In bony tissue, osteoclasts (derived from hematopoietic stem cells) and osteoblasts (of mesenchymal derivation) degrade and build and thus constantly remodel bone. Osteoclasts begin degrading bone, and the resorption pits are partly filled by a new bone matrix produced by osteoblasts, which is subsequently mineralized.

Bone is a very dynamic tissue, subject to continuous renewal to maintain homeostasis through bone remodeling, a process promoted by two cell types. Osteoblasts, of mesenchymal derivation, are responsible for the deposition of new material, and osteoclasts are hematopoietic cells, responsible for bone resorption. Osteoclasts differentiate from monocytes through the action of two cytokines, macrophage colony stimulation factor (M-CSF), and the receptor of activated NF-κB ligand (RANKL) which, once bound to their specific receptors on monocytes, induce differentiation in osteoclasts [1]. M-CSF is essential to ensure cell survival, while RANKL acts more specifically as a differentiation-promoting factor. After RANKL-RANK binding, tumor necrosis factor receptor-associated factor (TRAF) 6 is recruited. This leads to a series of signaling pathways (such as NFATc1, NF-kB, Akt/PKB, JNK, ERK and p38) with pivotal roles in osteoclast differentiation, function and survival [2]. In the body, the continuous bone remodeling (Figure 1) ensures the repair of microfractures and the compensation of wear due to biomechanical use, generally ensuring complete renewal in 7–10 months [3].

The skeletal system is extremely sensitive and fully capable of responding to local and systemic inputs. Pituitary and thyroid hormones, as well as sex hormones, for example, support the activity of osteoblasts, while cytokines can have different effects on the differentiation and activity of osteoblasts and osteoclasts. Many diseases are accompanied by an increase in bone resorption which in some cases can be attributed to hormonal deficiencies and/or aging [4], while others may give rise to systemic forms of bone loss that result in osteolysis. This is the case of inflammatory processes induced by autoimmune diseases such as rheumatoid arthritis, which cause chronic inflammation, arthroplasty interventions or bacterial infection [5], or some cancers that tend to affect bone tissue, leading to osteoblastic (typical of prostate cancer) or osteolytic metastases (typical of breast, lung and kidney cancers). Tumor growth in bone disrupts the delicate balance between resorption and formation and the local interaction of tumor cells with bone tissue forms a vicious cycle that facilitates the development of bone metastasis. In recent years there have been several studies on bone biology and the interactions between the skeletal and immune systems, and this has become an important research field—*osteoimmunology* [6]. Bone and the immune system are closely linked because bone regulates the hematopoietic stem cells from which all the immune system cells derive, while many immunoregulatory factors, such as interleukins (ILs), tumor necrosis factor-α (TNF-α) and the RANKL/RANK/OPG (osteoprotegerin) system also act on the differentiation of bone cells. The expression of RANKL in particular, among other genes, is a peculiar trait of the osteoblast lineage. Osteoblasts can also produce OPG, which acts as a decoy receptor, binding and blocking RANKL to inhibit osteoclasts activation and thus protect against bone loss [7]. Several studies have in fact proposed these new osteoimmunological biomarkers as useful tools in different conditions, for instance in assessing the progress of osteolytic bone metastases [7] or after total knee replacement surgery, to quantify the osteointegration of the prosthesis [8].

## 2. Osteomyelitis

Osteomyelitis (OM) is an invasive infectious process affecting bone or bone marrow [9] with severe compression of bone homeostasis, resulting in osteolysis. It is classified according to the etiological agent (pyogenic, mycobacterial, fungi, etc.) and the route of infection (hematogenous dissemination through the bloodstream; for contiguity). Osteomyelitis may also come from an infection in the surrounding soft tissue that spreads to the bone, from direct inoculation during trauma or medical interventions or from the anatomical position (tibia, femur, etc.) and duration (acute and chronic OM).

Hematogenic OM is the most common in children and almost 60% of cases are caused by *Staphylococcus aureus* [10]. OM has a higher incidence in males than females. Despite treatment, 30% of bone infections become chronic [11]. In adults, OM frequently affects patients with diabetes, trauma or after orthopedic surgery [12]. Hip and knee replacement surgery is the most common cause and bacterial biofilm formation on the foreign material is the major obstacle in its treatment [13].

Once the bone is infected, polymorphonuclear leukocytes (PMN) enter the site of infection, attempting to phagocytose infectious organisms. In order to destroy them, activated PMN releases several highly reactive oxidants which may damage bone tissue, resulting in lysis of the bone [14]. The pus spreads into the blood vessels of the bone, altering the flow and creating devitalized areas of infected bone, with consequent bone avascular necrosis and the formation of “sequestrum” (dead bone) which forms the basis of chronic infection. Often the body tries to create new bone around the necrotic area [14].

Chronic infection is due to bacteria, the most common cause being *S. aureus* [10]. Although other pathogens have been isolated, some in relation to age: in children the infection depends mainly on *Escherichia coli*, while in adults other gram negatives are more frequent (*Klebsiella* spp., *Enterobacter* spp. and *Pseudomonas* spp.). Osteomyelitis caused by fungi, virus or parasite infections is much less frequent. Tibia, femur, humerus, vertebrae and jaw are particularly sensitive to hematogenous OM because of their ample blood supply [15].

### 2.1. S. aureus and Osteoblasts

*S. aureus* can interact directly with osteoblasts both externally and internally as a result of its internalization [16]. *S. aureus* is equipped with several components on its surface, such as the cell wall peptidoglycans, lipoteichoic acid and lipoproteins, collectively defined as pathogen-associated molecular patterns (PAMPs). PAMPs can interact with osteoblasts, inducing the production and release of chemokines (CXCL2, CXCL8, CXCL10, CCL2, CCL3, CCL5) and cytokines (IL-1β, IL-18, TNF-α) that recruit and activate the innate (neutrophils, monocytes/macrophages) and adaptive (lymphocytes) immune cells [17,18]. In addition, *S. aureus* can inhibit de novo bone formation by preventing the expression of crucial markers of osteoblast growth and division such as alkaline phosphatase, collagen type I, osteopontin and osteocalcin. *S. aureus* also induces the secretion of soluble RANKL from osteoblasts, with consequent osteoclast recruitment and activation, leading to strongly imbalanced bone homeostasis [19,20] and inflammatory bone loss.

Ogawa was the first to demonstrate that *S. aureus* internalized into the osteoblast [21]. The main mechanism process is the link between fibronectin binding proteins A and B (Fnbp A/B)-fibronectin, serving as a bridge between *S. aureus* and osteoblasts through α5β1 integrin [22].

By internalizing into the osteoblast, *S. aureus* protects itself from the immune system in order to support and spread the infection. Inside the osteoblast, *S. aureus* is located in endolysosomal vesicles within which it can survive for a long time. This ability is due largely to the fact that, not being professional-phagocytic cells, the osteoblasts lack an effective and specific arsenal of antibacterial defense. This results in escape from these vesicles and release into the cytoplasm. *S. aureus* is in fact equipped with several membrane-damaging factors, among which the phenol soluble modulins (PSMs) stand out. PSMα, whose pro-escape action from the vesicles has been amply demonstrated [23,24,25], belongs to this family. PSMs promote bacterial release from the vesicles but also act on the cell membrane, compromising its integrity.

*S. aureus* can also survive for quite long times inside the osteoblast, as demonstrated by the presence of small colony variants (SCV). These are a bacterial subpopulation with an atypical, characteristic morphology, which makes it less aggressive for the host cell in order to ensure long survival inside. However, these long survival rates do not ensure the osteoblast’s safety. In the long run, in fact, the extremely harmful effects of PSMs on the cell membrane are lethal and still lead to the death of the osteoblast [25].

The osteoblast, however, does not remain passive in the face of attack by *S. aureus* but releases a whole series of inflammatory factors with the intent to participate in the innate antibacterial immune response that coordinates with the adaptive one mediated by Th1 lymphocytes [26]. In other words, it activates a complex interplay between the immune and bone systems that is included in the definition of osteoimmunology.

However, as already mentioned, the battle between *S. aureus* and osteoblasts is more or less lengthy, in favor of *S. aureus*, with consequent bone loss. *S. aureus* can inhibit osteoblast activity and differentiation and prevent mineralization [20,27,28,29,30].

*S. aureus* can also cause osteoblast apoptosis. This happens because of the activation of several pathways that lead to the same result [27,28,31,32,33]. Certainly, the osteoblast’s death is one of the most characteristic events of OM caused by *S. aureus* and has a double valence. On the one hand, the formation of new bone is reduced and on the other, the release of the bacterium from dead cells is promoted, facilitating the infection of surviving osteoblasts and exacerbating the infectious event [34].

### 2.2. S. aureus and Osteoclasts

Bone loss in OM does not depend solely on the loss of activity and/or the osteoblast’s death. Another very important factor is fundamental: the strong increase in the differentiation and activity of the osteoclasts. In this context, we must remember that osteoblasts produce two important factors in the regulation of osteoclastogenesis—RANKL and OPG.

RANKL binds to a specific receptor (RANK) on the precursor cells of osteoclasts. This interaction gives rise to the transformation of mononucleate precursors into mature osteoclasts. OPG, which is present in soluble form, acts as a decoy receptor for RANKL, limiting the bond with RANK to regulate osteoclastogenesis [35].

In the case of *S. aureus* infection, osteoblasts increase the production of RANKL and reduce that of OPG, increasing the rate of osteoclastogenesis [20]. It has also been shown [36] that osteoclasts infected with *S. aureus* give way to massive production of PGE_2_, a molecule capable of up-regulating the production of RANKL after binding to its specific EP_4_ receptor. The successful PGE_2_-EP_4_ bond leads to a further and massive increase in RANKL, greatly accelerating the osteoclastogenesis rate [37].

*S. aureus* (and/or its components) can also stimulate the release of proinflammatory cytokines such as TNF-α, IL-6 and IL-1β, and these too can enhance osteoclastogenesis by inducing the differentiation and activation of osteoclasts from the preosteoclast. In addition, these mediators induce the differentiation of monocytes and macrophages to pre-osteoclasts [38], further accelerating the formation of osteoclasts and aggravating the pathology [39].

These cytokines were significantly increased in both tissue and the circulatory system in animal models in which OM was induced, in human bone samples, and in patients’ plasma [40,41,42]. This, therefore, indicates not only a local but also a systemic involvement of these cytokines that are produced by different cell types and then released both locally and systemically. In addition, IL-6 or other mediators such as CCL2 (monocyte chemotactic-1 protein, MCP-1), CCL3 (macrophage inflammatory protein-1 alpha MIP-1α) and CXCL-2 (MIP-2) can be secreted from osteoblasts in response to exposure to *S. aureus* [43].

Hypothetically, the macrophages residing in bone—referred to as osteomacs—may act as additional triggering factors for OM-related inflammation [43]. Therefore one can reasonably assume that the osteoblasts and/or osteomacs are the initial source of proinflammatory mediators who will then promptly recruit the immune system cells, including macrophages, at the site of infection, resulting in ample production of cytokines in later stages of infection.

An important study by Truillet-Assant et al. [44] highlighted the point that the effect of S.aureus infection on bone-marrow derived osteoclast precursors depends on the differentiation status of the cells: if the infected cells are already committed to the osteoclast lineage, there will be an increase in RANKL-mediated osteoclast formation; otherwise, osteoclast differentiation will be inhibited, leading to the creation of an activated phenotype of macrophages M1 [45].

Neutrophils are the first line of immune defense against many bacterial infections and have an important role in OM. In this pathology, in fact, there is a significant increase in circulating neutrophils that accumulate at the site of bone infection, where bacteria are present. Proinflammatory cytokines such as IL-1 and TNF-α, released by neutrophils [46], cause activation of the inducible isoform of nitric oxide synthase (iNOs or NOS-2), resulting in the production of nitric oxide (NO) [47]. The presence of bacteria also leads to a more abundant production of NO due to the increased activity of the eNOS [48].

NO, produced in such amounts, boosts the catabolic rate of bone matrix proteins, induces osteoclastogenesis and bone resorption [47,49] and suppresses the differentiation of osteoblastic and marrow stromal cells [50].

Massive production of NO leads to a state of strong oxidative stress (OS) which can cause severe damage to biological macromolecules (such as nucleic acids, proteins and lipids) and to bone tissue and cartilage [51,52]. NO interacts with the superoxide anion radical to form peroxynitrite, a very strong oxidant that can cause various harm such as DNA damage and irreversible protein modification [53,54,55].

### 2.3. S. aureus and Osteocytes

Osteoblasts that have exhausted their function remain trapped in gaps in the bone matrix, produced by themselves, thus becoming osteocytes. Osteocytes are irregularly shaped cells with cytoplasmic processes extending away from the cell toward other osteocytes, in small channels called canaliculi. Through these canaliculi, nutrients and waste products are exchanged to maintain the viability of the osteocyte. Osteocytes, therefore, provide for the maintenance of the mineralized matrix thanks to the action of enzymes produced by them, and can reversibly remove minerals and reshape the organic phase of the bone matrix, a process described as osteolysis [56].

How this remodeling is involved in OM is still under investigation; however, induction of metalloproteinase expression has been observed in human osteocytes infected with *S. aureus* [57], suggesting that it affects osteolysis.

It has also been reported that human osteocyte-like cultures exposed to *S. aureus* presented robust induction of the expression of a large number of chemokines and cytokines [57], such as CXCL9, CXCL10 and CXCL11; this suggests active participation of osteocytes in the recruitment of cytotoxic and/or suppressive T-lymphocyte subsets to the infected sites [58].

## 3. Reactive Oxygen Species

ROS are a set of various reactive molecules and free radicals produced by mitochondria, because of electrons escaping during the process of oxidative phosphorylation, and other different sources, such as NADPH oxidases. ROS form a complex system of redox agents that actively intervene in the regulation of multiple cellular processes such as proliferation, metabolism, repair processes, apoptosis, differentiation, and migration [54]. ROS are therefore molecules with a dual role: useful when operating in cellular signaling, harmful when their levels rise unregulatedly because of factors such as aging, inflammation or age-related diseases such as osteoarthritis. This uncontrolled increase has lethal consequences for the cell [59,60,61]. ROS also regulate the function and differentiation of osteoclasts [62,63,64,65].

### 3.1. Superoxide Anion Radical

Superoxide is produced in large quantities by the enzyme NADPH oxidase (NOX), for use in the mechanisms of oxygen-dependent elimination of pathogens. In response to an inflammatory stimulus, NADPH oxidase catalyzes the transfer of electrons derived from the shunt of pentose phosphate, from the donor in the cytoplasm (NADPH) to the acceptor in the phagosome or in the extracellular space, oxygen (O_2_) allowing the formation of superoxide anions (O_2_^−^). O_2_^−^ is a precursor of many other ROS radicals responsible for the oxidative stress seen in several pathological processes, such as postmenopausal or diabetic osteoporosis and osteoarthritis [66].

O_2_^−^ is involved in the osteoclastic resorption resulting from the activation of osteoclasts by cytokines [67] and in fact, its accumulation is considered an indicator of the osteoclast activity.

### 3.2. NADPH Oxidase

NADPH oxidase is a multimeric enzyme complex present in different cell populations, known as one of the main causes of the production of ROS. This enzyme has seven isoforms (NOX1, NOX2, NOX3, NOX4, NOX5, DUOX1 and DUOX2) with different subcellular localizations [68]

*NOX1* is expressed mainly in the colonic epithelium though in other cells as well, including osteoclasts. NOX1 is also present in bone marrow macrophages (BMMs) where, despite its low level, it seems to have a role. Indeed, silencing it leads to a significant decrease in the production of ROS and inhibits osteoclast differentiation [63]. However, its contribution to bone turnover is still not entirely clear. Other studies [69] have shown that the knockdown of NOX1 alone did not lead to any decrease in the production of ROS, unlike a combined knockdown of NOX1 and NOX2, which is the main isoform in BMMs.

This has led to the hypothesis of combined action of the two NOX isoforms, able to compensate each other so as to avoid damage to the osteoclastogenesis [54].

*NOX2* is strongly expressed in macrophages and neutrophils where it generates O_2_^−^ indispensable for the elimination of pathogens [54]. NOX2, however, is the main isoform in osteoblasts and BMMs. Its activity is very important in the differentiation of osteoclasts. In fact, the NOX2-produced superoxide anion radical amplified RANKL-induced NFATc1 expression in osteoclast signaling [70]. The activity of NOX2 is tightly managed by a negative regulatory mechanism operated by the negative regulator of ROS (NRROS). It is therefore possible to hypothesize that NRROS increases during osteoclast differentiation until the NOX2 expression is suppressed, with the result of blocking osteoclastogenesis [71].

*NOX4* is expressed more in osteoclasts than in precursors, suggesting an important role in osteoclastogenesis and bone homeostasis. Loss of NOX4 activity leads to a stop in O_2_^−^ production and bone resorption [72]. Consequently, NOX4 knockout mice had higher bone density and fewer osteoclasts and bone resorption markers [73]. However, in the bone of patients with increased osteoclastic activity NOX4 expression was increased. Similarly, women carrying a single nucleotide polymorphism (SNP) positively associated with NOX4 expression had high levels of circulating markers of bone turnover and reduced bone density [65]. Thus NOX4 may offer a potential therapeutic target for the treatment of osteoporosis [73].

### 3.3. Hydrogen Peroxide

H_2_O_2_ is produced spontaneously or by superoxide dismutase 2 and 1 (SOD2 and SOD1), which rapidly drive O_2_^−^ conversion in the mitochondrial matrix and in the cytoplasm, respectively [74]. H_2_O_2_ is involved in several physiological processes such as cell differentiation, proliferation or apoptosis [75,76]. It triggers osteoclast differentiation and resorption in animal models and in human bone marrow stromal cells [77]. It can also affect osteoblasts. Yao and colleagues showed that H_2_O_2_ has negative action on osteoblast proliferation, stimulating their apoptosis [78].

### 3.4. ROS Signaling in Bone Remodeling

Bone homeostasis strongly depends on the balance between bone formation and resorption [52]. This delicate balance is one of the first to fail with aging when bone resorption prevails over formation [79]. ROS are crucial in bone turnover, and over the years several studies have investigated the relationship between formation, reabsorption and the role played by the ROS in this context [64,65,80]. OS can alter bone remodeling by inducing an imbalance in favor of the osteoclasts’ activity (favoring the differentiation of pre-osteoclasts in osteoclasts) until the onset of metabolic bone diseases and/or skeletal system disorders, including osteoporosis, marked by low bone mineral density and loss of bone mass, resulting in extreme bone weakness and a strong predisposition to fractures [77,81].

ROS can also limit, or even block and stop, the activity and differentiation of osteoblasts destined for apoptosis, as well as osteocytes, thus favoring even more osteoclastogenesis [82,83,84].

Many factors mainly produced by osteoblasts and osteocytes regulate the activity of the two types of cells and, consequently, bone remodeling; among the most important are RANKL and OPG, already mentioned. Their expression is highly sensitive to increased oxidative status, with consequent RANKL up-regulation and OPG down-regulation through the activation of protein kinases (ERK1/2, JNK, etc.) and/or other factors which affect specific transcription factors [85]. RANKL promotes the differentiation and activity of osteoclasts by interaction with specific receptors (RANK) located on precursor monocytes/macrophages and directs its differentiation toward osteoclasts and median bone resorptions. OPG, instead, produced by activation of the signaling pathway Wnt/βcatenin, is a soluble receptor that can bind and block RANKL, acting as a decoy receptor and inhibiting osteoclast activity [86,87].

OS leads to blockage of osteoblast activation and OPG production, resulting in increased RANKL action and osteoclast differentiation and induction of activity. All this is evident from an increase in the RANKL/OPG ratio, a true indicator of the intensity of bone resorption [88]. Increases in the levels of this index, therefore, indicate an imbalance toward bone resorption processes not compensated by the adequate formation and are related to the pathogenesis of various skeletal diseases, including different forms of osteoporosis and bone diseases secondary to inflammation [87] such as OM [89].

**Figure 2:**
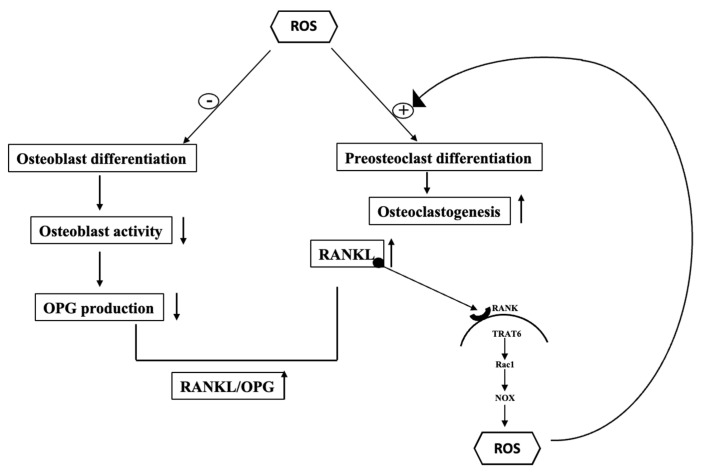
Effects of ROS on osteoblast and osteoclast differentiation/activity and RANKL/RANK pathway to ROS production.

Between OS and RANKL, almost a kind of self-powering circuit is created where the excessive production of ROS leads to blockage of osteoblast activation and OPG production, resulting in increased RANKL with the consequences previously examined. The RANKL, in turn, then promotes further production of ROS thanks to the participation of several molecules involved in intracellular signaling such as TRAF6; Rac1 and NOX [90,91]. TRAF6, though not directly involved in their generation, plays a key role in the production of ROS downstream of RANKL [63,64] thanks to its direct action on Rac1. Rac is a cytosolic component of the NOX complex and a downstream signal messenger of the Rho GTPase family. It has been reported to be involved in cytoskeletal organization and is responsible for the activation of NOX. The expression of a Rac1 dominant-negative mutant stops ROS production, thus indicating Rac1 acts directly in ROS generation [92]. Furthermore, considering the essential role of NOX in the production of ROS during the differentiation of osteoclasts [93], one can outline the signal cascade that leads from RANK to the production of ROS as follows: RANK, TRAF6, Rac1, NOX and ROS. A schematic representation of the effects of ROS and the related RANKL/RANK pathway is given in Figure 2.

## 4. Oxidative Stress Biomarkers in Osteomyelitis

Musculoskeletal infections (MSCI) such as OM are still not always straightforward to diagnose and treat. Timely diagnosis and hence appropriate treatment are essential; a delayed diagnosis, in fact, can result in substantial morbidity and devastating consequences throughout life, such as the destruction of joint cartilage or permanent physical disability [94,95].

Patients with OM often need intensive care in hospital; they have a high likelihood of severe multi-organ complications and require numerous surgical debridements [95,96]. It is therefore clear that prompt recognition of the OM is essential so as to start therapies as soon as possible and minimize the risk of consequences.

There have been many attempts with different markers to find those that are most sensitive and indicative. Van Asten and colleagues [97] examined a panel of inflammatory markers in order to diagnose and monitor OM in diabetic patients with a diabetic foot infection. The study included markers such as erythrocyte sedimentation rate (ESR), C-reactive protein (CRP), procalcitonin (PCT), IL-6, IL-8, TNFα, MCP-1 and MIP1α, following the course before therapy and during treatment. The results showed how inflammatory serum markers such as PCT can play a role both in detecting OM and in monitoring the course of therapy given the significant declines in CRP levels, ESR, PCT and IL-6 on continued therapy.

Another study worth mentioning was by Mo et al. [98]. With the aim of defining a novel panel of biomarkers and cytokines to distinguish septic arthritis from OM, they identified one set of markers that could differentiate the two pathologies in their initial stages using serum alone. Among the markers examined, CTx-II, a marker of cartilage breakdown, also associated with markers of bone metabolism [99] and MCP-I, an inflammatory chemokine, showed strong correlations with OM; this confirmed reports of high MCP-I in OM animal models and in OM patients [100,101].

Over-production of ROS is a recognized critical factor in several pathological bone disorders such as diabetic osteoporosis, rheumatoid arthritis, and osteolysis [65,102,103]. Excessive accumulation of ROS can trigger bone destruction events both because of the lower levels of antioxidant enzymes and a block of the differentiation of osteoblasts. The same ROS can at the same time accelerate bone resorption by osteoclasts, resulting in a reduction in trabecular bone mass [104].

As previously mentioned, one of the characteristic features of OM is a strong state of oxidative stress, as witnessed by many studies [54,105,106]. It follows that assessing their degree in an increasingly accurate and timely way should be useful in the management of the disease. In the literature, there are in fact several studies aimed at evaluating new, easy-to-measure and broadly available biomarkers that may be used, for example, as markers of a more or less severe prognosis.

The activity of serum paraoxonase (PON1); a calcium-dependent hydrolase glycoprotein, distributed among organs such as the intestine, liver and kidney, as well as in plasma [107] has been seen to decrease in diseases involving a strong state of oxidative stress, including knee osteoarthritis [108]. Koruk et al. investigated the behavior of these enzymes in subjects with OM [109].

They found a strong condition of oxidative stress, indicated by the high values of LOOH (serum lipid hydroperoxides) and ceruloplasmine and by a significant decrease in PON1 activity. In addition, considering that PON1 also has a pivotal role in protection against bacterial endotoxins through detoxification of lipid peroxides [110] and that OM is a bone inflammation due to bacterial infection (e.g., Staphylococcus aureus) and/or exposure to bacterial toxins [111], Koruk suggested that the low PON1 activity might be related to the drastic loss in protection against bacterial endotoxins, and subsequently to a far worse prognosis.

Other studies also suggest that reduced PON1 activity, low SH concentrations and high LOOH concentrations may play a role in the severity of different diseases, such as coronary atherosclerosis [112]. Koruk, therefore, concluded that the overall picture of the patients might indeed indicate a much more severe prognosis.

Grbic and colleagues [106], based on Koruk’s study, examined a set of oxidative stress markers such as hydroperoxides and malondialdehyde (MDA), total antioxidant activity (AOA), total vitamin C, ascorbic acid (Asc) and reduced/oxidized vitamin C ratio in 137 patients with acute OM.

The study, conducted throughout the duration of clinical treatment, showed that at recruitment all the OS markers were significantly altered, indicative of a strong OS situation, and suggesting that more than with a drop in vitamin C, OS manifested itself with the rise in the ratio of oxidized to reduced vitamin C (shift of the vitamin C redox status toward oxidized forms). The intensity of OS gradually returned to normal during treatment, in line with the clinical course of the disease.

Once again, it is clear that OS markers can be a valuable help in defining the framework of the infection and in monitoring therapy.

Jyoti [55] and colleagues focused on a series of markers such as vitamin C, SOD, reduced glutathione (GSH) and ceruloplasmine (Cp). Cp is a group of serum proteins whose levels rise as a result of tissue injury and/or infection [112,113,114]. Acting as an antioxidant, Cp eliminates free oxygen radicals in a similar way to SOD. Cp keeps iron in the oxidized ferric state thus preventing it from undergoing the redox cycle (ferric (Fe^+3^) to ferrous (Fe^+2^)) necessary for bacteria to start their toxic effects. Bacteria need iron in the ferrous state to be pathogenic. In this way, Cp inhibits bacterial cell growth.

The study pointed to strong OS in patients with chronic OM, as borne out by the high levels of all the markers: serum MDA, serum protein carbonyl and serum nitrite. This OS led to a compensatory rise in Cp in patients, underscoring its role as an antioxidant in chronic OM. On the basis of these results, Jyoti [55] suggested the usefulness of giving these patients an antioxidant together with conventional drugs to prevent or reduce oxidative damage and deterioration of the musculoskeletal tissues.

Another important point is oxidative damage to DNA. ROS can attack the DNA causing oxidative damage such as modifications to the bases and sugars and breakages of the single or double chain. One of the biomarkers most widely used in situations of OS is 8-hydroxy 2-deoxyguanosine (8-OHdG), formed by oxidative modification of a guanosine base [115]. The 8-OHdG is corrected by DNA repair proteins such as DNA glycosylase-1 (OGG1) and excreted in the urine. However, excessive oxidation base formation is seen in various diseases and complications. In addition, peripheral blood 8-OHdG has been reported to be associated with mutation and cancer [116].

Ozkan’s work was the first to examine the oxidative damage to DNA in OM [117]. Ozkan’s study confirmed that in patients with OM there was a strong state of OS as evidenced by the decline in markers such as SOD, catalase (CAT) and GSH and by the increase in MDA. The most interesting results, however, are related to 8-OHdG, which was significantly high in OM subjects and which proved to be significantly correlated with the other OS markers examined. More specifically, 8-OHdG levels had a significantly negative correlation with SOD, CAT and GSH. Furthermore, there was a positive correlation between MDA and 8-OHdG.

These data confirmed, on the one hand, the severity of the OS in these subjects and on the other, led the authors to suggest that oxidative DNA damage may increase the risk of complications in OM patients. In addition, they proposed the use of palliative therapies in OM to reduce oxidative DNA damage.

Once again, research and evaluation of new OS biomarkers that can be useful in the prognosis and follow-up of chronic OM are needed.

To conclude the discussion, another aspect should be addressed. It is claimed that the systemic damage caused by OS can be severe and deleterious; it is therefore not surprising that over the years research has focused on the efficacy of novel compounds with therapeutic potential for osteoclast-related diseases. Several pharmacological antioxidants have proved crucial in the fight against cellular stress resulting from the deleterious effects of ROS in several bone diseases. Three of these compounds, which mainly act by inhibiting NOX complex activation and ROS production, and which have been tested on animal and human models, merit particular attention (Table 1):

Alliin is indicated for the treatment of osteopenia [118]. Alliin (S-allyl-l-cysteine sulfoxide, SACSO) is the main component of aged garlic extract (AGE) and has broad-spectrum natural antioxidant properties. Alliin has a dual effect: it inhibits osteoclastogenesis (by blocking the c-Fos-NFATc1 signaling pathway) and it reduces the production of ROS, down-regulating the expression NOX1.

Apocynin, indicated for the treatment of osteoporosis [119], effectively reduces the level of ROS by inhibiting the assembly of NADPH oxidase [120].

EWHA-18278, indicated for osteoporosis and osteopenia [121], is a pyrazole derivative with high inhibitory potency on NOX isozymes. Blocking the activity of NOX, EWHA-18278 inhibits the responses of BMMs to RANKL, including ROS generation.

## 5. Conclusions

This review summarizes the nature and main causes of OM, focusing on the very important role of OS in the disease and its consequences on the delicate balance between osteoblastogenesis and osteoclastogenesis. We have therefore emphasized the possibility of measuring the level of OS more accurately as a valid support in the management of the disease.

Therefore this review highlights the need to find new OS markers that are more sensitive and easier to use. This need has been emphasized in the many studies in the literature, aimed at the evaluation of new, easy-to-measure and broadly available biomarkers that may be useful for the prognosis and follow-up of chronic OM.

It is clear, in conclusion, how closely related to this is the parallel development of new compounds to fight cellular stress resulting from the deleterious effects of ROS in different bone diseases; for new and more effective therapies.

## Figures and Tables

**Table 1:** Pharmacological compounds, which mainly act by inhibiting NOX complex activation and ROS production.

Bone Disease	Target	Pharmacological Compounds
Osteopenia	NOX/ROS	Alliin
Osteoporosis	NOX/ROS	Apocynin
Osteopenia/Osteoporosis	NOX/ROS	EWHA

## Data Availability

The data are contained within the article.
